# “Sandwich technique” enables preservation of hearing and antivertiginous effect in cholesteatomatous labyrinthine fistula

**DOI:** 10.1007/s00405-021-06934-y

**Published:** 2021-06-18

**Authors:** Anna Bartochowska, Marta Pietraszek, Małgorzata Wierzbicka, Wojciech Gawęcki

**Affiliations:** Department of Otolaryngology, Head and Neck Surgery, Poznan University of Medical Sciences, 49 Przybyszewskiego Street, 60-355 Poznan, Poland

**Keywords:** Labyrinthine fistula, Cholesteatoma, Intratemporal complication, Vertigo

## Abstract

**Purpose:**

The aim of the study was to assess hearing, surgical and clinical results of the treatment in patients with cholesteatomatous labyrinthine fistula (LF) focusing on the different techniques and materials used in the management.

**Methods:**

Study group included 465 patients. Cases with LFs discovered or confirmed during surgical procedure were thoroughly analyzed.

**Results:**

LFs were noted in 11.4% of all cases. Thirty-eight patients, with all follow-up data available, were included into the further analysis. Most LFs were located in the lateral semicircular canal (87%). LFs were assessed as small in 2 cases, as medium in 24 patients while 12 were described as large. Based on Dornhoffer and Milewski classification, 50% of LFs were classified as IIa, 24% as IIb, 6 LFs were very deep (type III), while 4—superficial (type I). The size and type of LF did not influence postsurgical complaints (*p* = 0.1070, *p* = 0.3187, respectively). Vertigo was less frequent in LFs treated by “sandwich technique”, especially those with opened endosteum. In 30 (79%) patients, hearing improved or did not change after surgery. Hearing outcomes were significantly better in the ears operated by means of CWU technique (*p* = 0.0339), in LFs with intact membranous labyrinth (*p* = 0.0139) and when “sandwich technique” was performed (*p* = 0.0159). Postsurgical bone conduction thresholds levels were significantly better in LFs covered by “sandwich method” (*p* = 0.0440).

**Conclusion:**

“Sandwich technique” (temporal fascia–bone pate–temporal fascia) enables preservation of hearing as well as antivertiginous effect in patients with cholesteatomatous labyrinthine fistula.

## Introduction

Labyrinthine fistula (LF) is the most common intratemporal complication of chronic otitis media with cholesteatoma. According to the literature, it concerns 4–12% of the ears operated due to this pathology [1, 2]. It mostly affects lateral semicircular canal (87%) and rarely promontorium (8%), superior (6%) and posterior part of the labyrinth (2%) [2]. Preoperative detection of LF based on symptoms and clinical examination is often not specific and not sensitive enough [3]. High-resolution computed tomography (HRCT) significantly improves these indicators allowing proper diagnosis in 85–100% cases [3–5]. However, management of this pathology still remains controversial. Despite years of disputes on the best method of treatment providing both closure of the fistula and preservation of hearing, otosurgeons constantly debate whether ‘‘to leave the matrix over fistula or to remove it completely’’ [1, 2], which approach—canal wall up (CWU) or canal wall down (CWD)—should be used [2, 3, 5], if intraoperative steroid administration improves the results [6, 7]. As far as we know, only one group of researchers [7] analyzed the impact of the technique of the fistula closure on the final outcomes.

The aim of the study was to assess hearing, surgical and clinical results of the treatment in patients with cholesteatomatous labyrinthine fistula focusing on the different techniques and materials used in their management.

## Materials and methods

Our study group included all patients operated on in our department due to cholesteatoma in the years 01.2015–03.2020 (465 altogether). We selected those with labyrinthine fistula discovered or confirmed during surgical procedure. These cases were thoroughly analyzed (patients hospitalized in 2015–2017 retrospectively while those treated in 2018–2020 mostly prospectively).

The following variables were collected: age, gender, comorbidities, signs and symptoms, otosurgical history, pre- and postoperative air and bone conduction thresholds (at 0.5, 1, 2 and 3 kHz), fistula characteristics (location, size and type), HRCT scans of the temporal bone, surgical technique (canal wall up versus canal wall down approach; method of LF closure), and perioperative steroid use.

Fistula size was described according to the Sanna et al. [8] who divided them into small (0.5–1 mm), medium (1–2 mm) and large (> 2 mm). Fistula-type assessment based on Dornhoffer and Milewski classification are [6]: I—fistula with erosion of the bony labyrinth with an intact endosteum; II—fistula with opened endosteum but intact membranous labyrinth (IIa—with undisturbed perilymphatic space; IIb where perilymphatic spaces were disturbed either by active accidental suctioning before recognition of the fistula or by ingrowth of the cholesteatoma); and III—fistula with open perilymphatic space with concomitant involvement or destruction of the underlying membranous labyrinth.

Cholesteatoma location, extent and advancement were described basing on the European Academy of Otology and Neurotology/Japanese Otological Society joint consensus staging system [9].

HRCT scans of the temporal bones were assessed preoperatively both by radiologists and otosurgeons.

**Fig. 1:**
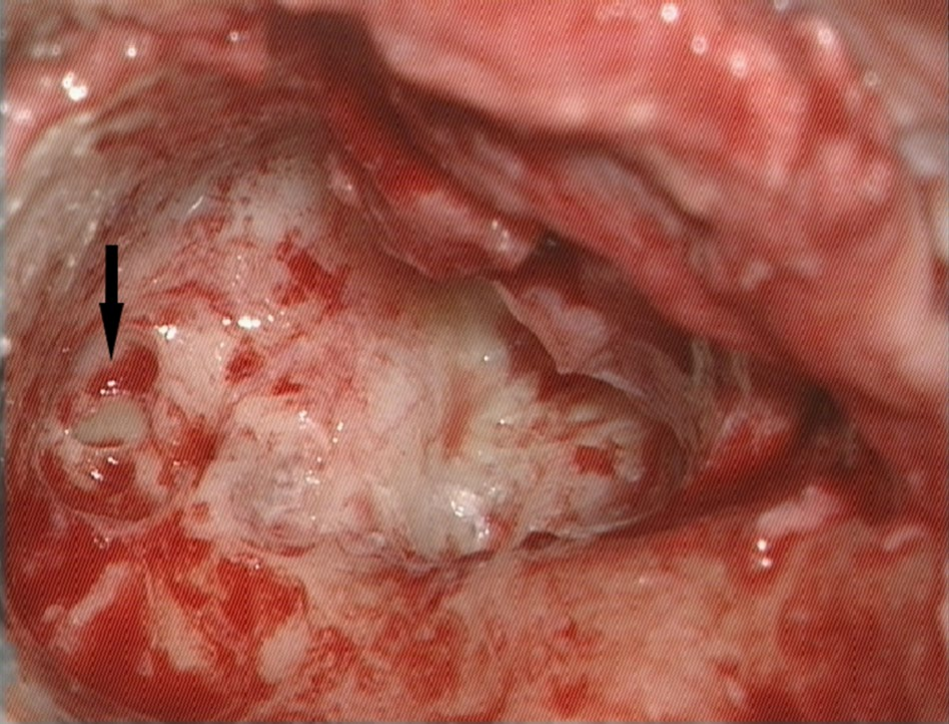
LF with opened endosteum before the closure

**Fig. 2:**
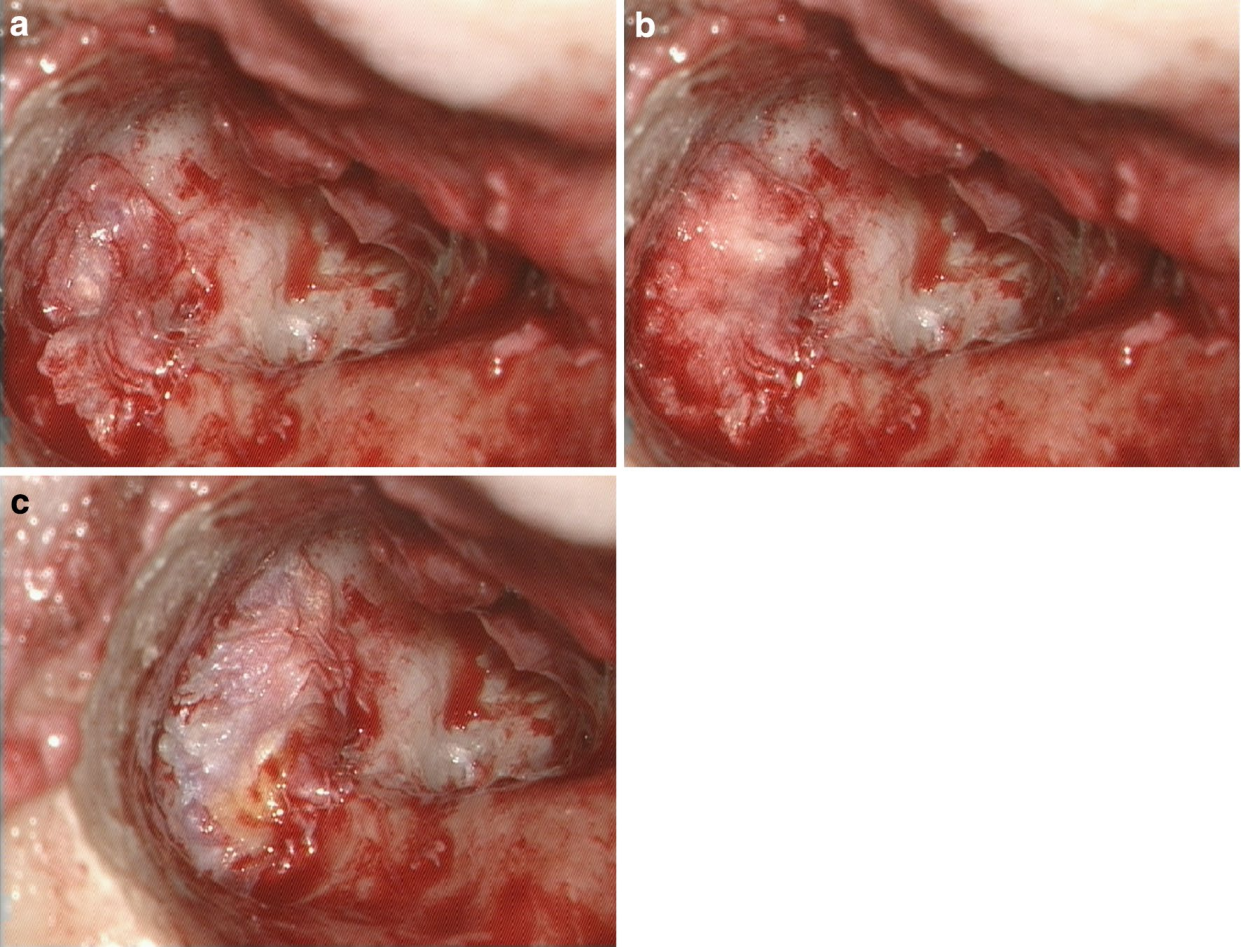
“Sandwich technique” as a method of LFs treatment, **a** first layer: temporal fascia, **b** second layer: bone pate, **c** third layer: temporal fascia)

All surgeries were performed by two experienced otosurgeons. The choice of surgical approach was independent of the presence of the fistula. It was mostly determined by cholesteatoma characteristics, patient’s general state and past ear history. CWD approach was usually chosen in cases with extensive, rapidly recurrent pathology, in difficult anatomical conditions as well as in patients with contraindications to perform another surgery in general anesthesia. In all cases with LF, removal of the cholesteatoma matrix from the fistula was performed after complete dissection of the pathology from the middle ear spaces. Since 2016 in most LFs mesna was additionally used to facilitate LF cleansing (a small amount (1–3 ml) of 20% saline solution of mesna was administered topically for ca. 2 min). We did not drill nor suction at the site of LF. We also did not perform labyrinthectomy or obliteration of LF. In all cases, cholesteatoma matrix was completely removed from the fistula in one-stage procedure. LF was immediately covered by autogenous material collected and prepared before the dissection of the matrix from the region of LF. The method of the fistula closure was mostly dependent on LF stage. In most type I LFs, bone pate was used, while in types II and III, bone pate and temporal fascia. In 2018, we started to perform “sandwich technique” in most cases of LFs with opened endosteum (Figs. 1 and 2a–c). A small layer patch of fascia was placed over the open fistula, then bone pate and again temporal fascia to stabilize two previous sheets. Intraoperative steroids were introduced into the treatment scheme of LF in our department in 2016. Sixteen milligrams of dexamethasone were given intravenously by anesthesiologists at the time of cholesteatoma removal. In all patients from the study group, steroids were administered intravenously after surgery at the ward (8 mg of dexamethasone twice a day for at least 3 days).

All patients were regularly followed up. In patients operated with CWU technique with no clinical signs of recurrent disease, non-echoplanar diffusion-weighted imaging magnetic resonance (MRI DWI NON EPI) was done 1 and 2 years after the surgery. In patients with recidivistic disease, reoperations were performed.

For statistical purposes, in the assessment of hearing outcomes, air and bone conduction thresholds from the audiograms performed at least 10 months after the surgery were considered.

Statistical analysis was conducted to determine factors influencing treatment outcomes. We focused on the method of fistula closure. Because of small sample size and more than 20% of cells with expected frequencies less than 5, we used Fisher Exact test for comparison of variables. All calculations were performed at the level of alpha = 0.05 using R software.

The study was approved by our institutional ethics committee.

## Results

LF was noted in 53 out of 465 analyzed patients which constituted 11.4% of all cases operated on due to the cholesteatoma in our department. Thirty-eight cases, with all follow-up data available, were included into the further analysis.

### Epidemiological data/comorbidities

There were 20 women and 18 men. Mean age of patients at the time of surgery was 47 years (range 11–76 years). Twelve patients suffered from hypertension, three were treated for diabetes, while one for hypothyroidism, one for asthma and one for chronic kidney disease (Table 1).

**Table 1:** Clinical characteristics of patients with labyrinthine fistula (LF)

Mean age (years)	47.32 ± 16.40 SD
	Number of patients (%)
Sex	
Female	20 (52.63)
Male	18 (47.37)
Comorbidities	
Hypertension	12 (31.58)
Diabetes	3 (7.89)
Hypothyroidism	1 (2.63)
Asthma	1 (2.63)
Chronic kidney disease	1 (2.63)
Signs and symptoms	
Ear discharge	26 (68.42)
Hearing loss	34 (89.47)
Vertigo	18 (47.37)
Clinical examination	
Positive fistula sign	12 (32.58)
Nystagmus	7 (18.42)
Otosurgical history	
Primary surgery	20 (52.63)
Second procedure	9 (23.68)
History of 2 or more procedures	9 (23.68)
Cholesteatoma location in the middle ear (STAM)	
Difficult-to-reach recesses (S):	
Protympanum (S1)	11 (28.95)
Sinus tympani (S2)	14 (36.84)
Tympanic cavity (T)	20 (52.63)
Attic (A)	26 (68.42)
Mastoid process (M)	36 (94.74)
Cholesteatoma advancement	
I. One location	–
II. Two or more locations	–
III. With extracranial or intratemporal complications	37 (97.37)
IV. With intracranial complications	1 (2.63)
Hearing results	
Conductive hypoacusis	10 (26.32)
Mixed hypoacusis	25 (65.79)
Deafness prior to surgery	3 (7.89)

### Preoperative clinical data

Most patients reported symptoms typical for cholesteatoma: ear discharge (68%) and hearing loss (89%). Vertigo and dizziness were present in 18 cases—in 12 patients, the course of the disease was gradual while in 6—with rapid onset. In only 12 cases, a positive fistula sign was found. In 7 patients, nystagmus was examined, in 2 beating towards the healthy ear.

Primary surgery was performed in 20 cases. Eighteen patients have been operated before (10 in other departments), out of which 9 more than once.

In 10 patients, conductive hearing loss was detected, in 25—mixed hypoacusis while 3 ears were deaf prior to surgery. The preoperative mean bone conduction level was 29 dB while mean air threshold was at the level of 57 dB.

All preoperative clinical data are presented in Table 1.

Based on HRCT the presence of fistula was described by radiologists in 26 cases and detected preoperatively by otosurgeons in 30 imagings.

### Intraoperative findings

Most LFs were located in the lateral semicircular canal (LSC) (87%), in 2 patients—in superior semicircular canal (SSC), while in 3—LFs were multiple (in LSC and SSC). LFs were assessed as small in 2 cases, as medium—in 24 patients while 12 were described as large. Based on Dornhoffer and Milewski classification, 50% of LFs were classified as IIa, 24% as IIb, 6 LFs were very deep (type III), while 4—superficial (type I) (Table 2).

**Table 2:** LF characteristics

	Number of patients (%)
LF location	
LSC	33 (86.84)
SCC	2 (5.26)
Multiple LFs	3 (7.89)
LF size	
Small	2 (5.26)
Medium	24 (63.16)
Large	12 (31.58)
LF type	
I	4 (10.52)
IIa	19 (50.00)
IIb	9 (23.68)
III	6 (15.79)

*LF* labyrinthine fistula, *LSC* lateral semicircular canal, *SCC* superior semicircular canal

In 8 cases, LF was detected intraoperatively (patients had no symptoms neither HRCT showed the presence of LF).

In 53% of cases, cholesteatoma was located in the tympanic cavity (T), in 68% in attic (A) and in 95% in mastoid process (M). In 14 patients, sinus tympani (S2) was involved, in 11 protympanum (S1). Most cholesteatomas (97%) were evaluated as stage III (Table 1).

There were found no statistically significant differences between cholesteatoma location and advancement and fistula size (*p* = 0.9999, *p* = 0.9999, respectively) and type (*p* = 0.1833, *p* = 0.5000, respectively).

### Surgical management

CWU technique was chosen in 19 cases. CWD was also performed in 19 patients (out of whom 10 have been operated by CWD method previously). In 15 cases, mesna was additionally used. In all the ears, there were no signs of residual cholesteatoma in neither middle ear spaces nor fistula region at the end of surgery. In 12 cases, bone pate was used to close LF, in 9—bone pate and temporal fascia, while in 17 “sandwich technique” was performed. Intraoperative steroids were used in 20 cases. In all the patients, intravenous steroids were prescribed in postoperative time and given for 3–7 days (mean 5 days).

### Postoperative outcomes

Postoperatively, ten patients (26%) presented vertigo (which lasted for 3–30 days)—six of them reported dizziness before the surgery as well. In all these cases, LFs were medium or large, in 3 cases—type IIb, in 3—type III, while in 4—IIa. The size and type of LF did not influence postsurgical complaints (*p* = 0.1070, *p* = 0.3187, respectively). Although it is not confirmed in statistical analysis (*p* = 0.1604), we noted that postoperative vertigo was less frequent in LFs treated by “sandwich technique”, especially those with opened endosteum.

In 30 (79%) patients, hearing (defined as a summary mean air and bone conduction gain) improved or did not change after surgery. In 6 cases, air and bone conduction threshold levels lowered (mean hearing loss: 25 dB for air and 10 dB for bone conduction). Two patients presented postoperative deafness. In all LFs treated with “sandwich technique”, hearing was preserved or improved. Hearing outcomes were significantly better in the ears operated by means of CWU technique (*p* = 0.0339), in LFs with intact membranous labyrinth (*p* = 0.0139) and when “sandwich technique” was performed (*p* = 0.0159). Postsurgical bone conduction threshold levels (defining inner ear function) were significantly better in LFs covered by “sandwich method” (*p* = 0.0440) (Table 3).

**Table 3:** Treatment outcomes

	Postoperative vertigo (%)		p*	Postsurgical inner ear function (BC thresholds) (%)			p*	Hearing outcomes (%)			p*
	Yes	No						Improved	Unchanged	Worsened	
				Improved	Unchanged	Worsened					
LF size											
Small	0 (0.00)	2 (5.26)	0.1070	0 (0.00)	2 (5.26)	0 (0.00)	0.7413	1 (2.63)	1 (2.63)	0 (0.00)	0.8062
Medium	4 (10.53)	20 (52.63)		2 (5.26)	20 (52.63)	2 (5.26)		8 (21.05)	11 (28.95)	5 (13.16)	
Large	6 (15.79)	6 (15.79)		0 (0.00)	10 (26.32)	2 (5.26)		2 (5.26)	7 (18.42)	3 (7.89)	
LF type											
I	0 (0.00)	4 (10.53)	0.3187	0 (0.00)	4 (10.53)	0 (0.00)	0.7325	1 (2.63)	3 (7.89)	0 (0.00)	0.0139
IIa	4 (10.53)	15 (39.47)		2 (5.26)	16 (42.11)	1 (2.63)		10 (26.32)	6 (15.79)	3 (7.89)	
IIb	3 (7.89)	6 (15.79)		0 (0.00)	7 (18.42)	2 (5.26)		0 (0.00)	5 (13.16)	4 (10.53)	
III	3 (7.89)	3 (7.89)		0 (0.00)	5 (13.16)	1 (2.63)		0 (0.00)	5 (13.16)	1 (2.63)	
Method of treatment											
CWU	4 (10.53)	15 (39.47)	0.7140	2 (5.26)	16 (42.11)	1 (2.63)	0.3477	9 (23.68)	8 (21.05)	2 (5.26)	0.0339
CWD	6 (15.79)	13 (34.21)		0 (0.00)	16 (42.11)	3 (7.89)		2 (5.26)	11 (28.95)	6 (15.79)	
Method of fistula closure											
Bone pate	4 (10.53)	8 (21.05)	0.1604	0 (0.00)	11 (28.95)	1 (2.63)	0.0440	4 (10.53)	5 (13.16)	3 (7.89)	0.0159
Bone pate + fascia	4 (10.53)	5 (13.16)		0 (0.00)	6 (15.79)	3 (7.89)		1 (2.63)	3 (7.89)	5 (13.16)	
“Sandwich technique”	2 (5.26)	15 (39.47)		2 (5.26)	15 (39.47)	0 (0.00)		6 (15.79)	11 (28.95)	0 (0.00)	
Other surgical data											
Reoperations	5 (13.16)	13 (34.21)	0.9999	0 (0.00)	17 (44.74)	1 (2.63)	0.3506	5 (13.16)	8 (21.05)	4 (10.53)	0.9999
Mesna	3 (7.89)	12 (31.58)	0.7085	1 (2.63)	13 (34.21)	1 (2.63)	0.9999	5 (13.16)	8 (21.05)	2 (5.26)	0.7502
Intraoperative steroids	4 (10.53)	16 (42.11)	0.4681	1 (2.63)	18 (47.37)	1 (2.63)	0.6546	6 (15.79)	10 (26.32)	3 (7.89)	0.8309

*BC* bone conduction, *LF* labyrinthine fistula, *CWU* canal wall up, *CWD* canal wall down

*p Fisher exact test

The mean follow-up time in the study group was 23 months (range 10–48 months). In 3 patients, residual disease was detected in MRI DWI NON EPI, in other 3 cases recurrence was diagnosed in otoendoscopy. In all recidivistic ears, cholesteatoma was confirmed in reoperations. There were no cases of recurrent LFs in the study group.

## Discussion

The management of LF has been a subject of debate for years. In the literature, there is still the lack of consensus in the treatment schemes. In fact, optimal guidelines do not exist.

The first point of controversy still regards the technique (CWU or CWD) that should be performed in case of cholesteatomatous LF. Years ago, most otosurgeons recommended radical or modified radical mastoidectomy as the only methods allowing sufficient visualization of LFs and their proper management [10, 11]. Nowadays, most authors underline that the decision on preservation of posterior canal wall should not be determined by the presence of LF but should depend on many factors including hearing status of the operated and contralateral ear, location and extension of the cholesteatoma, coexistence of other complications, patient’s age and general condition [3, 5, 12]. However, when we analyze results presented in the latest literature, it turns out that CWD seems to be preferred technique in LF cases (Table 4) [1, 5, 13–16]. While in some departments CWD is still the method of choice [15], detailed analysis shows that most surgeons choose the approach that primarily enables them radical removal of the cholesteatoma. Although in our study group, 50% of patients were treated by CWD technique, we underlined that the choice of the method was dependent on cholesteatoma related factors and was not determined by the presence of LF itself. What important, almost 50% of our patients have been operated before (26% by CWD) which could probably pave the way for LF development (too strenuous drilling in the region of LF during the previous procedures?) or indicate the aggressive pattern of cholesteatoma growth with increased bone destruction processes. In our opinion, if possible, CWU approach should be used as the first-line treatment in all LF cases. Moreover, extreme caution regarding thorough HRCT analysis (also by otosurgeons regarding the fact that, if not directed by information in the referral form, radiologist can miss describing the presence of LF–—we had such a situation in several cases) and delicate surgical dissection of cholesteatoma in the region of semicircular canals should be maintained in all reoperated ears.

**Table 4:** Results presenting LF management and hearing outcomes published in the literature from the last 5 years and compared to our data

	Type of mastoidectomy	Cholesteatoma matrix management	Fistula management	Corticosteroids	Hearing outcomes (postoperative bone conduction)
Misale et al. 2019 [16]	NA	Removed completely (+ irrigation)	Bone wax + temporalis fascia Temporalis fascia + conchal cartilage + temporalis fascia	−	Unchanged/improved in 100%
Rosito et al. 2019 [15]	CWD 100%	Type II—complete removal, type III—partial retention	Temporal fascia and bone pate	+	Unchanged/improved in 75%
Baylan et al. 2018 [14]	CWD 91%	Removed completely	Temporal fascia, cartilage, bone pate	−	Mean values—unchanged/improved
Geerse et al. 2017 [12]	CWU 42%, PHORC 22%, subtotal petrosectomy 27%, radical mastoidectomy 9%	Removed completely in all cases	Fascia + fibrin glue	−	Unchanged/improved in 86%
Sagar et al. 2017 [1]	CWD 86%, radical mastoidectomy 14%	Removed completely in all cases	1 case—temporalis muscle and fascia Other—NA	−	Unchanged/improved in 92%
Meyer et al. 2016 [5]	CWD 81% (2 patients after CWD) with obliteration by Palva flap, CWU 19%	Removed completely in all cases	Fascia temporalis + bone dust (36%), bone dust + biologic glue (21%), bone dust after drilling the canal (17%), fascia temporalis (14%), cartilage + fascia temporalis (2%), temporal muscle + fascia temporalis (2%), temporal muscle + biologic glue (5%), temporal muscle + cartilage + biologic glue after drilling the canal (2%)	1 case	Unchanged/improved in 75,7%
Bo et al. 2016 [13]	Radical mastoidectomy 51%, CWD 46%, Bondy technique 3%	Removed completely	Temporalis fascia	−	Most patients—unchanged/improved
Our study	CWU (50%), CWD (50%)	Removed completely in all cases, in 53% mesna was used	Bone pate (31%), bone pate and temporal fascia (24%), “sandwich technique” (45%)	+ (39%)	Unchanged/improved in 89%

*LF* labyrinthine fistula

The second point of dispute concerns the issue of the removal of the cholesteatoma from the LF [1, 7]. Fortunately, the idea of leaving the matrix over LF was preferred years ago [8, 17]. Many studies show that the risk of hearing loss by labyrinthine damage during the dissection of cholesteatoma matrix from the LF area is equal when compared to the risk of its damage by collagenases present in residual epithelium [18]. Moreover, preservation of the cholesteatoma in the region of LF may lead to delayed complications like suppurative labyrinthitis or meningitis [10]. Therefore, precise dissection of the matrix from the fistula seems to be a mainstay of the proper treatment. In our study, radical removal of the cholesteatoma was performed in all cases. We paid attention to delicate preparing of the epithelium from LF, in most cases preceded by the use of mesna, without suctioning and drilling in its region. We think that such a protocol should be recommended in all LFs.

In the literature, we found only one article analyzing the impact of the material used in LF closure on the final outcomes. Gocea et al. [7] did not find the differences between conchal cartilage, bone dust, periosteum and the mixture of them on the clinical results. Other otosurgeons, except for the tissues listed above, preferred temporal fascia, temporal muscle, bone wax or biologic glue in different configurations. We are advocates of “sandwich technique” introduced in our department in 2018. Previously we tended to use bone pate or bone pate covered by temporal fascia. We noticed that patients in whom LFs with opened endosteum were sealed with bone pate presented more intense and longer dizziness than those in whom LFs were covered by temporal fascia first. It was probably caused by small bone particles that entered semicircular canals and behaved like free-floating otoliths. On the other hand, fascia alone, especially when used in CWD approach, had a tendency to relocate. It also seems to be insufficient material to close LF—patients after CWD with such a reconstruction very often experience vertigo when exposed to the cold wind while people treated by CWU method feel dizzy in situations of increased pressure in the middle ear. It encouraged us to close LFs with three layers—small patch of temporal fascia over the open fistula, then bone pate and again temporal fascia to stabilize two previous sheets. “Sandwich method” did not only reduce postoperative vertigo in our patients but also improved hearing outcomes. It was probably due to better closure of LF. Jang et al. [19] achieved good antivertiginous results by obliterating CWD mastoid cavities. They believed that obliterative mass can form a barrier that protects labyrinth from induced pressure changes. Our three sandwich layers probably acted the same.

In many articles, the protective role of intraoperative steroids on hearing is being raised [6, 7, 19]. They have antioxidative and antiapoptotic activity, antiproliferative effect on cholesteatoma regrowth in the LF site as well as the ability to stabilize intralabyrinthine membranes [6, 7, 20]. The method was introduced to LF management by Dornhoffer and Milewski [6]—they used intraoperatively 500 mg injection of methylprednisolone at the time of LF cleansing and observed beneficial impact of such a procedure on treatment outcomes in LFs with open labyrinth. Considering steroid induced side effects, we decided to use smaller doses—16 mg of dexamethasone which equals 85 mg of methylprednisolone. Although some authors [12] suggest that steroids do not improve hearing results, we think that they can have positive effect on that key treatment outcome.

Among methods facilitating removal of the matrix from the fistula laser [21], special techniques, e.g., underwater ear surgery [22] and mesna [23] are described. We do not have experience with the first mentioned method, but we appreciate significant role of mesna in atraumatic LF clearance. We recommend it in the management of cholesteatomatous LF as well.

## Conclusions

“Sandwich technique” (temporal fascia–bone pate–temporal fascia) enables preservation of hearing as well as antivertiginous effect in patients with cholesteatomatous labyrinthine fistula. Radical gentle removal of the matrix from LF, without suctioning and drilling in its region, facilitated by topical mesna use and intraoperative intravenous injection of steroids, followed by immediate covering of LF is a safe LF management protocol. The choice of surgical approach should not be determined by the presence of LF but should depend on hearing status of the operated and contralateral ear, location and extension of the cholesteatoma, coexistence of other complications, patient’s age and general condition. Extreme caution regarding thorough HRCT analysis as well as delicate surgical dissection of cholesteatoma in the region of semicircular canals should be maintained in all reoperated ears.

## Data Availability

Yes.

## Code availability

MS Excel.
